# First-principles study of two dimensional C_3_N and its derivatives†

**DOI:** 10.1039/d0ra06534j

**Published:** 2020-09-10

**Authors:** Zhao Chen, Haidi Wang, ZhongJun Li

**Affiliations:** School of Electronic Science and Applied Physics, Hefei University of Technology Hefei Anhui 230009 China haidi@hfut.edu.cn zjli@hfut.edu.cn

## Abstract

Here we have performed a comprehensive first-principles study for electronic and mechanical properties of newly synthesized C_3_N and its derivatives. The C_3_N monolayer is evaluated to be an indirect semiconductor with a HSE06 level bandgap of 1.09 eV, which can be effectively tuned by the number of layers, stacking order and B-doping concentration. With strong polar covalent bonds, C_3_N is predicted to be a superior stiff material with high in-plane Young's modulus (1090.0 GPa) and thermal dynamic stability (up to 2000 K). Remarkably, the C_3_N monolayer possesses a fascinating bending Poisson's effect, namely, bending induced lateral contraction, which is rare in other 2D materials. What's more, C_3_N nanosheets can be rolled into nanotubes with a tunable bandgap corresponding to the radius of curvature. Due to high stability, suitable band gap and superior mechanical strength, two dimensional C_3_N will be an ideal candidate in high-strength nano-electronic device applications.

## Abstract 2

Here we have performed a comprehensive first-principles study for electronic and mechanical properties of newly synthesized C_3_N and its derivatives.

## Graphical abstract

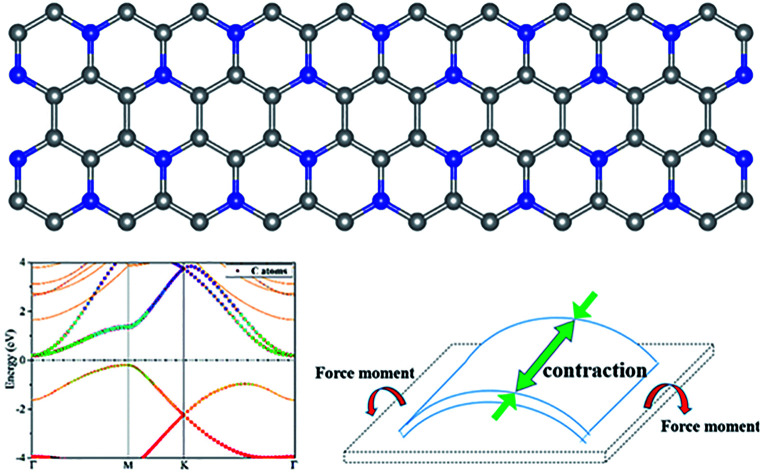

## 1 Introduction

Since the first two-dimensional (2D) material, namely graphene, was successfully fabricated by Novoselov and Geim,^1^ 2D materials research has rapidly risen to be one of the hot spots of condensed matter physics due to their fascinating electronic, mechanical, optical or thermal properties.^2–4^ For instance, many fantastic phenomena are discovered in graphene,^5^ due to the presence of a Dirac-type band dispersion. However, the gapless feature, at the same time, also limits its application in electronic and optoelectronic devices. Subsequently, few layer black phosphorus^6–8^ was successfully synthesized and it is predicted that it not only has a direct band gap of about 2.0 eV, but also has a high carrier mobility. Unfortunately, black phosphorene degrades readily when it exposes to the moist air.^9^ At the same time, due to the low Young's modulus, the black phosphorene is too flexible to be applied under high mechanical environment.^10^ Therefore, exploring new 2D semiconducting materials with high stability, strong mechanical strength and suitable band gaps are still a long-term target.

Recent years, a hole-free 2D crystal consisted of carbon and nitrogen atoms, named C_3_N, has been fabricated by polymerization of 2,3-diaminophenazine.^11^ C_3_N monolayer possesses an indirect band gap of 0.39 eV (PBE level) that can be tuned to cover the entire visible range by fabrication of quantum dots with different diameters. The back-gated field-effect transistors made of monolayer C_3_N display a high on/off ratio of 5.5 × 10^5^. More importantly, a experimental research designs an artificial synapse with tunable synaptic behavior based on solution-processed 2D C_3_N/polyvinylpyrrolidone (PVPy), which can mimic the synapse cleft based on proton conducting mechanism and may find further applications in artificial intelligence.^12^ Therefore, a further examining of the mechanical and electronic structure properties will be helpful to further exploit other applications of this new type of 2D material.

In this work, we firstly present the basic methodology that we used to in this simulation. Then detailed results of geometry structure, and electronic properties of layered C_3_N are discussed, including the mechanical properties of two-dimensional C_3_N and C_3_N-based nanotubes. Finally, we give conclusions and outlooks.

## 2 Computational methods

In this work, all the first-principles calculations are performed based on the Kohn–Sham density functional theory^13^ (KS-DFT) as implemented in the Vienna *Ab initio* Simulation Package^14^ (VASP). The generalized gradient approximation within the Perdew–Burke–Ernzerhof^15^ (PBE) functional form is used for the exchange–correlation energy. The plane wave basis sets with kinetic energy cutoff of 500 eV are used to expand the valence electron wave functions. For all structural relaxations, the convergence criterion for the energy in electronic SCF iterations and the Hellmann–Feynman force in ionic step iterations are set to be 1.0 × 10^−6^ eV and 5.0 × 10^−3^ eV Å^−1^, respectively. In order to reduce the interaction between neighboring layers, a large vacuum space of at least 15 Å is introduced along the *z*-axis. The Brillouin zone is represented by Monkhorst–Pack^16^ special *k*-point mesh of 12 × 12 × 1 for geometry optimizations, while a larger grid (16 × 16 × 1) is used for SCF computations. Besides, HSE06 hybrid functional is used to obtain an accurate band gap.^17^ van der Waals (vdW) correction proposed by Grimme (DFT-D2) is used due to its good description of long-range vdW interactions for multi-layered 2D materials.^18–22^ As a comparison, the Becke88 van der Waals^23^ (optB88-vdW) functional is also used for multi-layered structure. *Ab initio* molecular dynamics (AIMD) simulations with NVT ensemble are performed to assess the thermal stability of C_3_N monolayer.

## 3 Results and discussion

### 3.1 Geometry structure and electronic properties of layered C_3_N

**Fig. 1:**
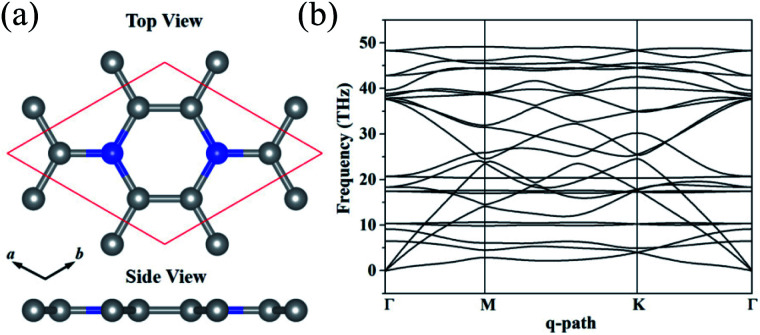
(a) Top and side view of monolayer C_3_N in the unit cell. (b) Phonon band structure C_3_N.

The optimized structure of C_3_N monolayer is shown in Fig. 1(a). The structure possesses *P*6/*mmm* symmetry (space group ID 191) with hexagonal lattice. The optimized lattice constants are *a* = *b* = 4.862 Å. The top view shows that the new phase is similar to graphene sheet, however, the six membered rings are composed by either C and N atoms or all C atoms. Unlike blue phosphorene,^24^ silicene^25^ and some other puckered materials,^26,27^ the C_3_N has a flat structure from the side view. In this structure, all C and N atoms are sp^2^ hybridized forming conjugated π bond. The C–C (1.40 Å) and C–N (1.40 Å) bond lengths show pronounced characters of single bonds. The unit cell of C_3_N contains 8 atoms as denoted by red box in Fig. 1(a) in which the C to N ratio is 3 : 1.

Before studying the electronic and mechanical properties of C_3_N, we firstly determine whether it is stable or not. To confirm the dynamical stability, the phonon dispersions of C_3_N are calculated by using the finite displacement method as implemented in PHONOPY.^28^ The calculated phonon dispersion curve in Fig. 1(b) shows no imaginary modes in the entire Brillouin zone, which confirms that C_3_N is dynamically stable. Moreover, we also carried out *ab initio* molecular dynamics (AIMD) simulation with 4 × 4 × 1 supercell to judge the thermodynamic stability of C_3_N monolayer. After heating at high temperature (1000 K) for 5 ps with a time step of 1 fs, no structure reconstruction is found. Furthermore study indicates that C_3_N can also withstand temperature under 2000 K. Remarkably, the melting point is getting close to 3000 K, suggesting that C_3_N monolayer has superior thermal stability (see in ESI Fig. S1†).

**Fig. 2:**
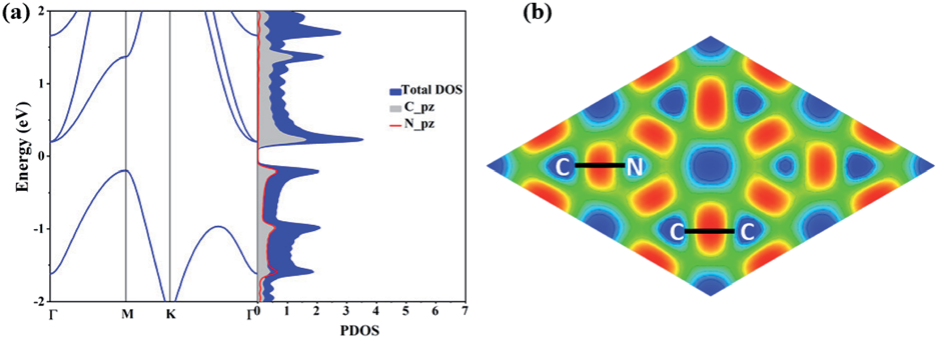
(a) Electronic band structure and projected density of state (PDOS). Γ (0.0, 0.0, 0.0), M (1/2, 0.0, 0.0) and K (−1/3, 2/3, 0.0) refer to special points in the first Brillouin zone. (b) The electron localisation function (ELF) of C_3_N.

The electronic structure properties of C_3_N are then investigated. The electronic band structure as well as density of states (DOS) of C_3_N monolayer are calculated under the PBE level of theory. As shown in Fig. 2(a), unlike gapless graphene,^29^ C_3_N is predicted to be indirect band gap semiconductor with a gap of 0.39 eV, as the valence band maximum (VBM) and the conduction band minimum (CBM) are located at the M point and the Γ point in the Brillouin zone, respectively. From the projected band structure and density of states (DOS), one can see that the VBM is a hybrid state of C-pz and N-pz, however, the CBM is mainly contributed by pz orbital of C atoms, which is consistent with their electronic charge density (see in ESI Fig. S2†). The calculated bandgap value is consistent with previous work.^11^ It is well-known that DFT within PBE level of theory underestimates the bandgap of semiconductors.^30^ Therefore, a more accurate hybrid functional HSE06 (ref. 17) is employed to correct the bandgap. We verify that the band dispersion profiles remain the same (see in ESI Fig. S2†), but the band gap value increases to 1.09 eV. By calculating the electron localization function (ELF),^31,32^ the C–C and C–N covalent bonds in C_3_N can be identified (Fig. 2(b)).

In addition, according to our Bader's charge analysis,^33^ each C atom transfers about 1.3*e* to N atoms, which implies a significant polarizability of C–N covalent bonds. Compared with previously proposed N chains encapsulated in carbon nanotubes^34^ (0.4*e*) and the penta-CN_2_ (ref. 35) (1.2*e*), the nitrogen atoms in C_3_N receive much more charge, indicating a much stronger interaction between C and N atoms, which to some extent indicates that C_3_N will hold high Young's modulus.

**Fig. 3:**
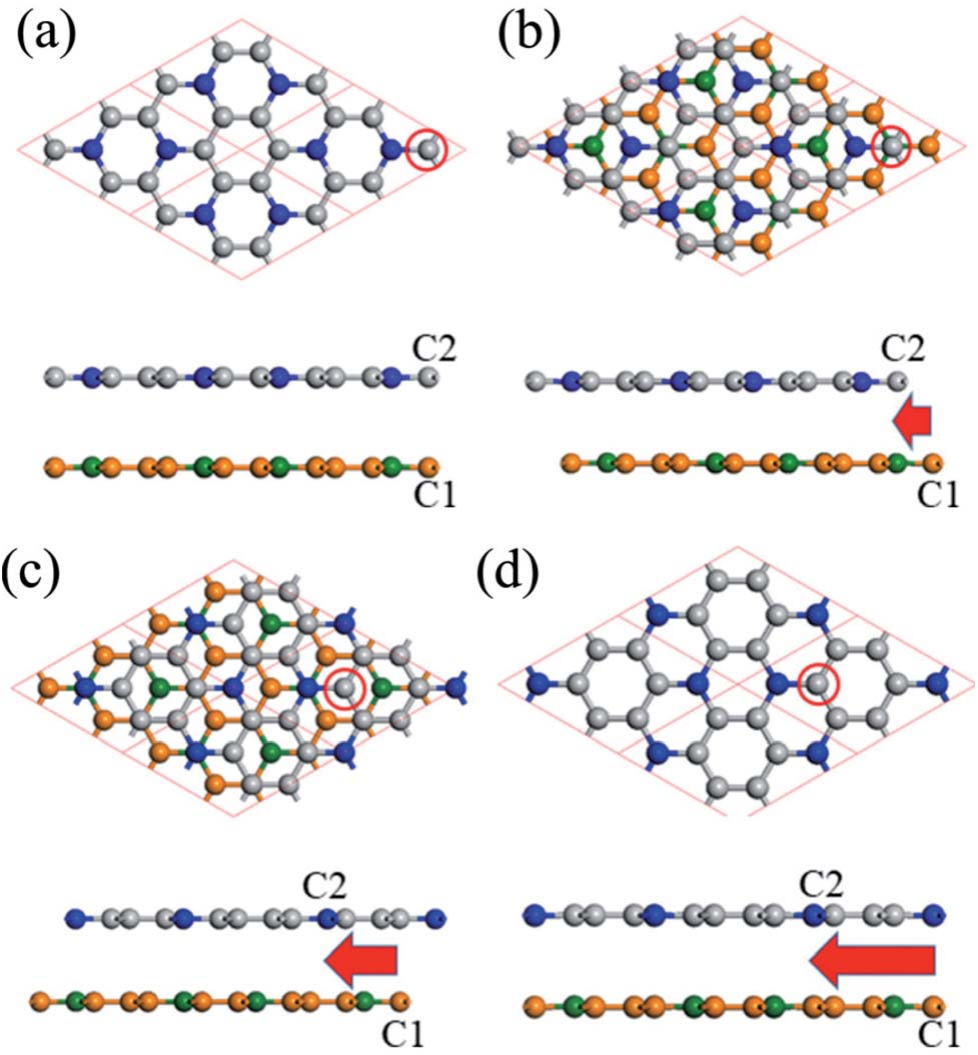
Four stacking structures of bilayer C_3_N, (a)–(d) top view and side view of AA-, AB-, AC- and AD-stacking, respectively. A 2 × 2 super cell is adopted for the top view. The red arrow indicates the relative shift of C1 and C2 atom. The C2 atom in the top layer is labelled by red circle. The C atoms in different layers are denoted by gray and yellow spheres. And the N atoms in different layers are denoted by blue and green spheres.

We also calculate the multi-layered C_3_N nanosheet. As for the geometric structure of bilayer C_3_N, we here consider four kinds of high symmetry stacking order, namely AA-, AB-, AC- and AD-stacking. As shown in Fig. 3a, in AA-stacking, the top layer is directly stacked on the bottom layer and they are matched perfectly in *xy*-plane. The AB-, AC and AD-stacking can be viewed as shifting the top layer of atoms along the vector *a*–*b* with a displacement as displayed in Fig. 3. As a comparison, the relative energy, layer distance and bandgap are listed in Table 1. We firstly optimize the structure by including DFT-D2 correction due to its good description of interlayer interaction. The calculated layer distance is in the range of 3.20–3.40 Å, which is close to the value of its parent material graphene (3.35 Å)^36^ and analogue C_2_N-h2D (3.18 Å).^37^ By analyzing the relative energy, we may find that AD-stacking is the most favorable configuration for bilayer C_3_N, being 0.093, 0.03 and 0.017 eV per unit cell lower than that of AA-, AB and AC-stacking, respectively.

**Table 1:** The relative energy, layer distance and bandgap of bilayer C_3_N under different functional

Stacking order	Relative energy eV per unit cell		Layer distance Å		Bandgap eV		
	DFT-D2	optB88	DFT-D2	optB88	DFT-D2	optB88	HSE06
AA	0.093	0.086	3.40	3.41	0.00	0.00	0.27
AB	0.003	0.007	3.22	3.25	0.22	0.23	0.83
AC	0.017	0.017	3.20	3.20	0.01	0.08	0.62
AD	0.000	0.000	3.23	3.21	0.00	0.00	0.48

In addition, the calculated relative energy and layer distance with optB88 vdW functional are consistent with the DFT-D2 ones, this further demonstrates the validity of our calculation. Due to the small energy difference, these configurations are expected to transform to each other under appropriate condition. Therefore, it is desired to investigate electronic structure of different stacking order. As listed in Table 1, the most stable configuration AD-stacking and metastable AA-stacking, the AB- and AC-stacking are predicted to be semiconductors with a HSE06 level bandgap of 0.48, 0.27, 0.83 and 0.62 eV, respectively. For trilayer C_3_N, we consider four kinds of high symmetry stacking structures based on the three stacking orders of bilayer C_3_N: ADA-stacking, ADB-stacking, ADC-stacking, and ADD-stacking. The results show that ADA-stacking is the most stacking order. On the whole, the electronic structure of multi-layered C_3_N is closely related to the number of layers and stacking order. The bandgap can be effectively tuned by the number of layers and stacking order, which supplies an optional way to tune electronic structure of C_3_N.

**Fig. 4:**
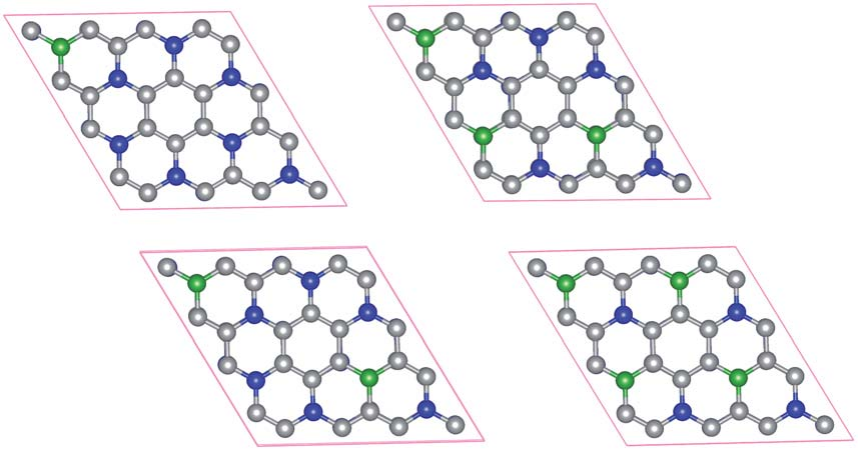
Structures of B doped bilayer C_3_N with 1, 2, 3 and 4 B-atoms for (i) case. The C atoms, N atoms and B atoms are denoted by gray, blue and green spheres.

Doping has been widely used to tune the electronic structures of 2D materials.^38,39^ In this work, we systematically study the electronic structures of bilayer C_3_N by B doping. Here we only consider the most stable configuration (AD-stacking) for B doping. A 2 × 2 × 1 supercell of bilayer C_3_N is adopted to analyze the B doping effects. There are eight N atoms in each layer of C_3_N. A hexagonal pattern of N atoms are substituted by the B dopant atoms, including two series of configurations. (i) Only one layer of bilayer C_3_N is doped with 1, 2, 3 and 4 B-atoms, corresponding to dopant concentrations of 6.25%, 12.5%, 18.75% and 25%, respectively (Fig. 4). (ii) Both layer of C_3_N is doped with 1, 2, 3 and 4 B-atoms, corresponding to dopant concentrations of 12.5%, 25%, 37.5% and 50%, respectively. Based on the PBE-level simulation with DFT-D2 correction, the band structures of bilayer B-doped C_3_N are obtained in Fig. S3.† For case (i), B-doped bilayer C_3_N with concentration of 12.5% is metal. The B-doped bilayer C_3_N with concentration of 6.25% is an indirect semiconductor with a bandgap of 0.023 eV, while 18.75% and 25% are direct semiconductors with a bandgap of 0.15 eV and 0.24 eV, respectively. For case (ii), B-doped bilayer C_3_N with concentration of 12.5% is metal. The B-doped bilayer C_3_N with concentrations of 25%, 37.5% and 50% are indirect semiconductors with a bandgap of 0.21 eV, 0.48 eV and 1.30 eV, respectively. In all, the bandgap of AD-stacking C_3_N can be tuned by B doping. Particularly, in case (ii), the bandgap of AD-stacking C_3_N increases as the doping rate increases, which makes C_3_N suitable for designing modern electronic devices.

### 3.2 Mechanical properties and C_3_N nanotubes

**Fig. 5:**
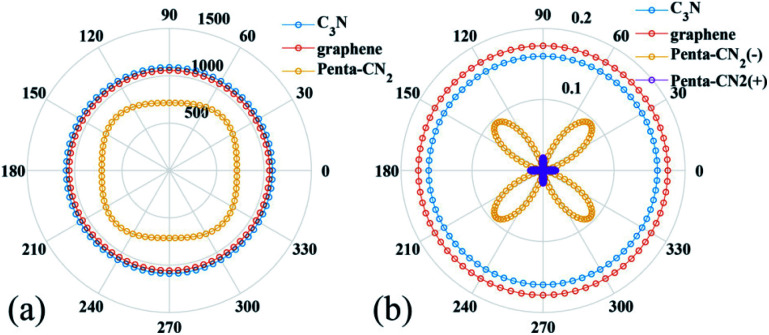
(a) Orientation dependent (a) Young's modulus (GPa) and (b) Poisson's ratio of C_3_N, penta-CN_2_ and graphene. Penta-CN_2_(−) and penta-CN_2_(+) indicate negative and positive Poission's ratio of penta-CN_2_, respectively.

By using the finite distortion method,^40^ the linear elastic constants of C_3_N monolayer are obtained (see in ESI Table S1†). Due to the symmetry of geometric structure, there are three independent elastic constants for 2D hexagonal crystal, namely, *C*_11_(*C*_22_ = *C*_11_), *C*_12_ and *C*_66_. The elastic constants of C_3_N satisfy the requirement of the Born criteria,^41^ namely *C*_11_ > 0, *C*_66_ > 0 and *C*_11_ − *C*_12_ > 0, which further demonstrates the mechanical stability of C_3_N. To have a deep knowledge of mechanical properties of C_3_N, we plot the orientation dependent Young's modulus and the Poisson's ratio according to eqn (S1) (see in ESI).† As comparison, the Young's modulus and the Poisson's ratio of some representative 2D materials, such as graphene^42,43^ and penta-CN_2_,^35^ are also plotted Fig. 5(a) and (b). Unlike penta-CN_2_, the Young's modulus and the Poission's ratio of C_3_N is isotropic due to its high symmetry. More important than the isotropic mechanical properties, the Young's modulus of C_3_N (1090.0 GPa) is comparable or a little higher than that of graphene (1057.7 GPa) and much higher than that of penta-CN_2_ (794.7 GPa), borophene (646.6 GPa)^44^ and black phosphorene (166.0 GPa),^10^ which suggests that 2D C_3_N is as stiff as graphene. To explore the ideally tensile strength (the highest achievable stress of a defect-free crystal at 0 K) and critical strain (the strain at which ideal strength reaches)^45^ of C_3_N, an in-plane biaxial tensile stress is applied. The stress–strain relationship for monolayer C_3_N is presented in Fig. S4 (see in ESI),† where the tensile strain ranges from 0 to 20%. The ideal strength under the critical strain (14%) is 207.1 GPa (equivalent value 66.5 N m^−1^), which is apparently larger than that of black phosphorene,^10^ δ-phosphorene,^46^ MoS_2_ (ref. 47) and borophene.^48^ From the high Young's modulus and large ideal strength, we may conclude that C_3_N monolayer is a stiff material.

To simulate the out-of-plane bending deformation of C_3_N monolayer, the C_3_N nanosheet is rolled into the corresponding nanotube, which has been widely studied in other 2D materials, such as graphene,^49,50^ boron nitride,^51^ blue phosphorene^52^ and black phosphorene.^53^ Here, monolayer C_3_N is directly rolled up to C_3_N-nanotube. Specifically, two types of nanotube is considered in this work, namely, zigzag (*n*, 0) for *n* = 3, 4, … 7 and armchair (*n*, *n*) for *n* = 2, 3, … 6 (see in ESI Fig. S5†).

**Fig. 6:**
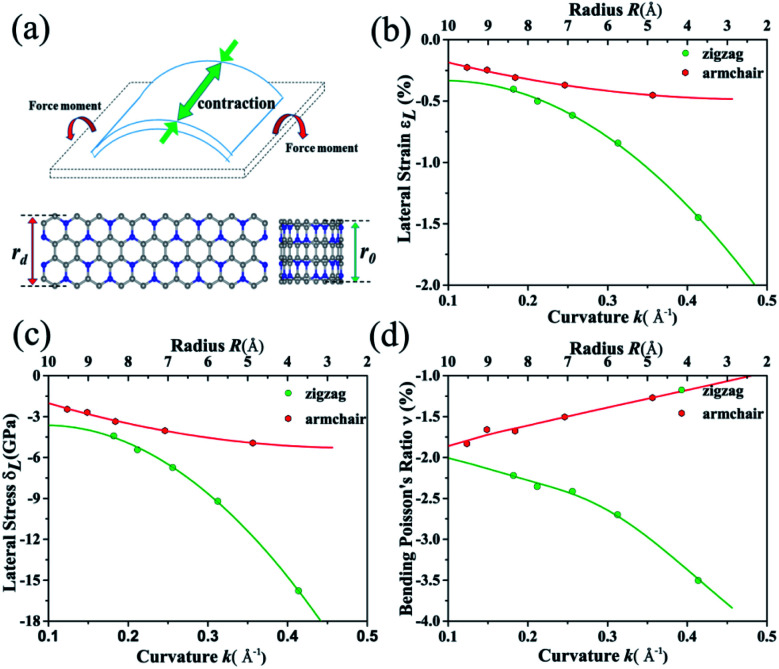
(a) Schematic plot of homogeneous elastic plate under a pure bending loading. The plates with and without mechanical load are depicted by the blue and black dashed line. C_3_N nanosheet and corresponding nanotube are also included. (b), (c) and (d) Bending induced lateral strain, equivalent lateral stress and bending Poisson's ratio as a function of curvature for bending along the zigzag (green line) and armchair (red line) directions.

According to the Poisson's ratio formulates, lateral strains in a material can be caused by a uniaxial stress in the perpendicular direction, but no net lateral strain should be induced in a thin homogeneous elastic plate subjected to a pure bending load. Here, we find that significant exotic lateral strains can be induced while bending C_3_N sheet to corresponding nanotube (see Fig. 6(a)). Taking zigzag nanotube as an example, the lattice constant *r*_0_ of all (*n*, 0) tube should be equal to the width of C_3_N sheet without bending load *r*_d_. However, as listed in Table S2,† all of lattice constants *r*_0_ are smaller than *r*_d_ = 8.422 Å, indicating that C_3_N monolayer has an interesting bending Poisson effect.^54^ To have deep knowledge of this behavior, the bending Poisson ratio *ν*, defined as the ratio of lateral strain to the curvature of C_3_N-nanotube (*ν* = *ε*_L_/*k*) is evaluated, where the lateral strain introduced by the bending load can be written as *ε*_L_ = (*r*_d_ − *r*_0_)/*r*_0_ and corresponding lateral stress is defined as *σ*_L_ = *Yε*_L_. *Y* is the in-plane Young's modulus of C_3_N monolayer. According to the *ε*_L_–*k*, *σ*_L_–*k* and *ν*–*k* relations illustrated in Fig. 6(b)–(d), some typical features of the bending Poisson's effect in C_3_N monolayer can be summarized: (i) the bending-induced lateral strain is orientation-dependent. For instance, when the radius of curvature down to 2.6 Å, the lateral strain along the armchair direction is −0.45%, while the zigzag direction is up to −1.45%, which should be easy to be detected by experiment. In addition, the zigzag nanotubes with small radius of curvature have a more obvious bending Poisson's effect than the armchair ones, however, with the increase of radius of curvature, the lateral strain of both armchair and zigzag nanotubes reduces to the 0. (ii) The variation trend of lateral stress under different radius of curvature is similar to that of lateral strain. Compared with in-plane strain–stress curve of C_3_N monolayer, the lateral stress is much smaller than in-plane strain-inducted equivalent stress. (iii) The bending Poisson ratio is a function of curvature. As for armchair nanotube, the *ν*–*k* relation is approximately linear, while zigzag nanotube has a nonlinear relation. To explain bending Poisson's effect, a local structure of C_3_N nanotube is shown in Fig. S6 (see in ESI†). It can be seen that the perfect C_3_N sheet has a flat structure, where all carbon and nitrogen atoms hold sp^2^ hybridization. However, when out-of-plane bending load is employed to the C_3_N monolayer, the sp^2^ hybridization states are destroyed. To accommodate the bending load, the N atoms will prone to form sp^3^ hybridization as it is in NH_3_ molecule. Finally, the C atoms linked the same N atom will get close to each other and lead to an axial contraction.

Finally, as a byproduct of bending effect investigation, the basic electronic and mechanical properties of C_3_N nanotubes are also investigated (see in ESI Table S2†). For both armchair and zigzag C_3_N nanotube, as expected, the strain energy decreases while *d* increases. The Young's modulus of all the nanotubes we selected are approximately equal to 1000.0 GPa except the (3, 0)-nanotube, which are consistent with recent work.^55,56^ It is worth noting that the Young's modulus of tubes is also comparable or a little higher than that of graphene. A qualitatively calculation shows that all of them are semiconductors with their bandgaps related with radius of curvature *d*. However, the zigzag nanotubes give rise to a much smaller band gap compared to that of the armchair nanotubes with nearly the same *d*. In detail, the band gap of (5,0)-nanotube is 0.97 eV, while that of (3, 3)-nanotube is up to 1.43 eV, which is even higher than that of flat C_3_N monolayer, indicating that bending load can be a tool to tune the band gap of C_3_N. In addition, we calculate the optical absorption for C_3_N nanotubes as displayed in Fig. S7.† C_3_N nanotubes show absorption in the visible-light and UV-light range. What's more, we can modulate the energy range of the absorption peak with different tubes. The absorption peak of the armchair tube always has a small blue shift relative to the zigzag tube. Considering the excellent properties of optical absorption for C_3_N nanotube, it is suitable for optoelectronic device.

## 4 Conclusions

In summary, we conduct a first-principles simulation to investigate the thermal and dynamic stability, electronic and mechanical properties of two dimensional C_3_N and its derivates. C_3_N monolayer is predicted to be a semiconductor with suitable bandgap, ultra-high mechanical strength and thermal stability, whose electronic properties can be tuned by layer number and stacking order. As for mechanical aspect, C_3_N possesses a fascinating bending Poisson's effect, resulting from the change of hybridization state of N atoms in local C_3_N skeleton. Besides, most of the corresponding nanotubes also present high Young's modulus and semiconducting properties, which may extend the application of C_3_N materials. Considering these high stability, superior mechanical strength and semiconducting properties of C_3_N and related derivative, two dimensional C_3_N is expected to have promising potentials to compete not only against graphene but also against other 2D materials for various applications, particularly in nanotransistors, fabrication of polymer nanocomposites with superior mechanical response. We hope our research can stimulate more experiments work on this subject.

## Conflicts of interest

There are no conflicts of interest to declare.

## Supplementary Material

RA-010-D0RA06534J-s001
