# Supplementary material for: First-principles study of two dimensional C3N and its derivatives
Source: RSC Adv. 2020 Sep 10;10(55):33469–74. doi: 10.1039/d0ra06534j (PMC9056723; doi:10.1039/d0ra06534j)
Supplement: RA-010-D0RA06534J-s001 [file RA-010-D0RA06534J-s001.pdf]

Electronic Supplementary Material (ESI) for RSC Advances.
This journal is © The Royal Society of Chemistry 2020

First-principles study of two dimensional C_3N and its derivatives†

Zhao Chen, Haidi Wang,* ZhongJun Li*

School of Electronic Science and Applied Physics,Hefei University of Technology, Hefei, Anhui 230009, China.

*Corresponding Author: E-mail: haidi@hfut.edu.cn, zjli@hfut.edu.cn

Equation

$$E(\theta) = \frac{Y_{zz}}{cos^4\theta + d_2 cos^2\theta sin^2\theta + d_3 sin^4\theta} \quad (1)$$

$$\nu(\theta) = \frac{\nu_{zz} cos^4\theta - d_1 cos^2\theta sin^2\theta + \nu_{zz} sin^4\theta}{cos^4\theta + d_2 cos^2\theta sin^2\theta + d_3 sin^4\theta} \quad (2)$$

where,

$$\nu_{zz} = \frac{C_{12}}{C_{22}} \quad (3)$$

$$d_1 = \frac{C_{11}}{C_{22}} + 1 - \frac{C_{11}C_{22} - C_{12}^2}{C_{22}C_{66}} \quad (4)$$

$$d_2 = -(2\frac{C_{12}}{C_{22}} - \frac{C_{11}C_{22} - C_{12}^2}{C_{22}C_{66}}) \quad (5)$$

$$d_3 = \frac{C_{11}}{C_{22}} \quad (6)$$

$$Y_{zz} = \frac{C_{11}C_{22} - C_{12}^2}{C_{22}}. \quad (7)$$

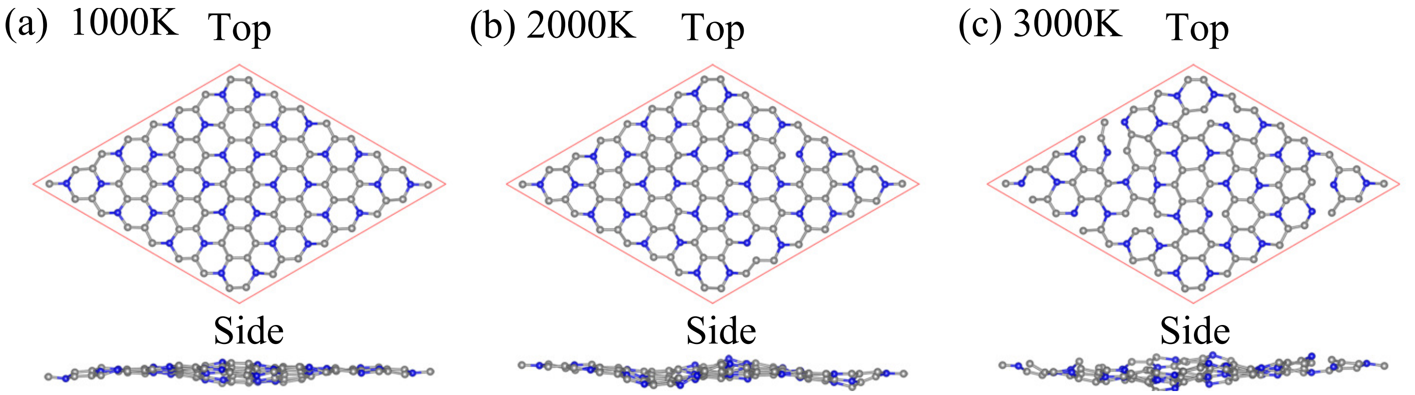

Figure S1 (a), (b) and (c) the snapshot of $C_3N's$ atomic configuration at the end AIMD simulation under 1000 K,2000 K and 3000 K, respectively

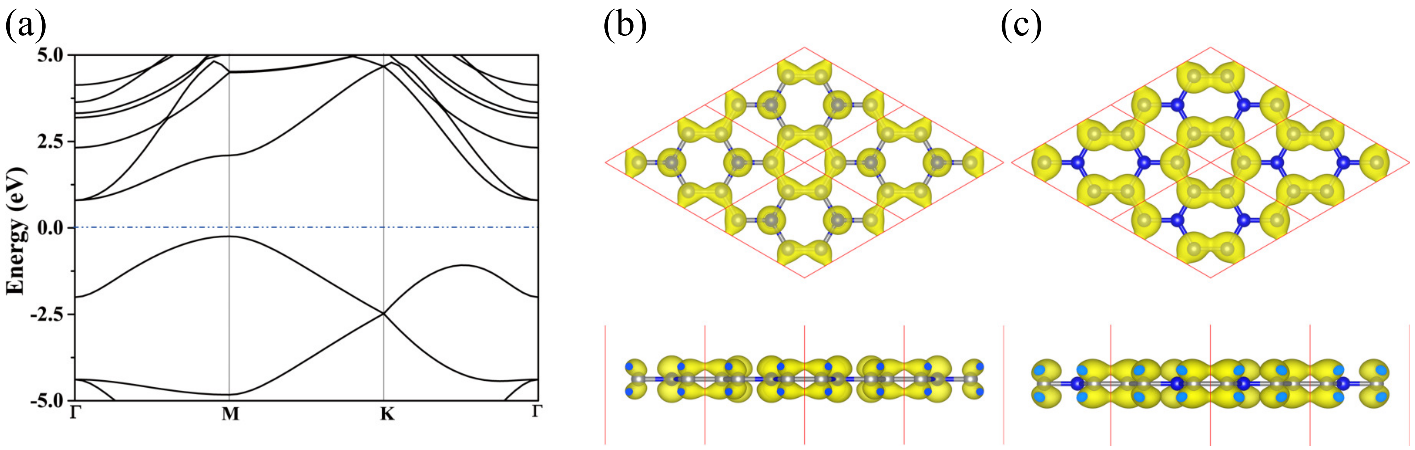

Figure S2 (a) Electronic band structure of C_3N with HSE06 level of theory.Electronic density distribution of (b) the valence band maximum and (c) the conduction minimum.

Table S1 Independent elastic constants (C_{ij}, GPa), Young's modulus (Y, GPa) and Poisson's ratio (ν) of C_3N, graphene and penta-CN_2 monolayer.

System	C_{11}	C_{22}	C_{12}	C_{66}	Y	ν
C_3N	1119.0	1119.0	180.1	469.4	1090.0	0.16
Graphene	1091.3	1091.3	191.6	449.6	1057.7	0.18
Penta-CN_2	714.0	714.0	12.9	438.4	713.7 to 794.7	-0.09 to 0.02

Table S2 The calculated diameter (d), lattice constant along the axial direction (r_0), bandgap under HSE06 level of theory (E_{gap}), Young's modulus (Y) and strain energy (δE) for C_3N nanotubes.

System	d(Å)	r_0 (Å)	E_{gap}(eV)	Y(GPa)	δE(eV/$atom$)
(3,0)	4.835	8.300	0.55	958.3	0.236
(4,0)	6.398	8.351	0.79	1010.9	0.138
(5,0)	7.814	8.370	0.97	1047.4	0.087
(6,0)	9.444	8.380	1.08	1045.8	0.060
(7,0)	11.006	8.388	1.14	1045.2	0.043
(2,2)	5.612	4.840	1.25	1046.4	0.214
(3,3)	8.123	4.844	1.43	1075.5	0.088
(4,4)	10.866	4.847	1.42	1071.2	0.046
(5,5)	13.446	4.850	1.38	1081.4	0.028
(6,6)	16.184	4.851	1.33	1080.5	0.019

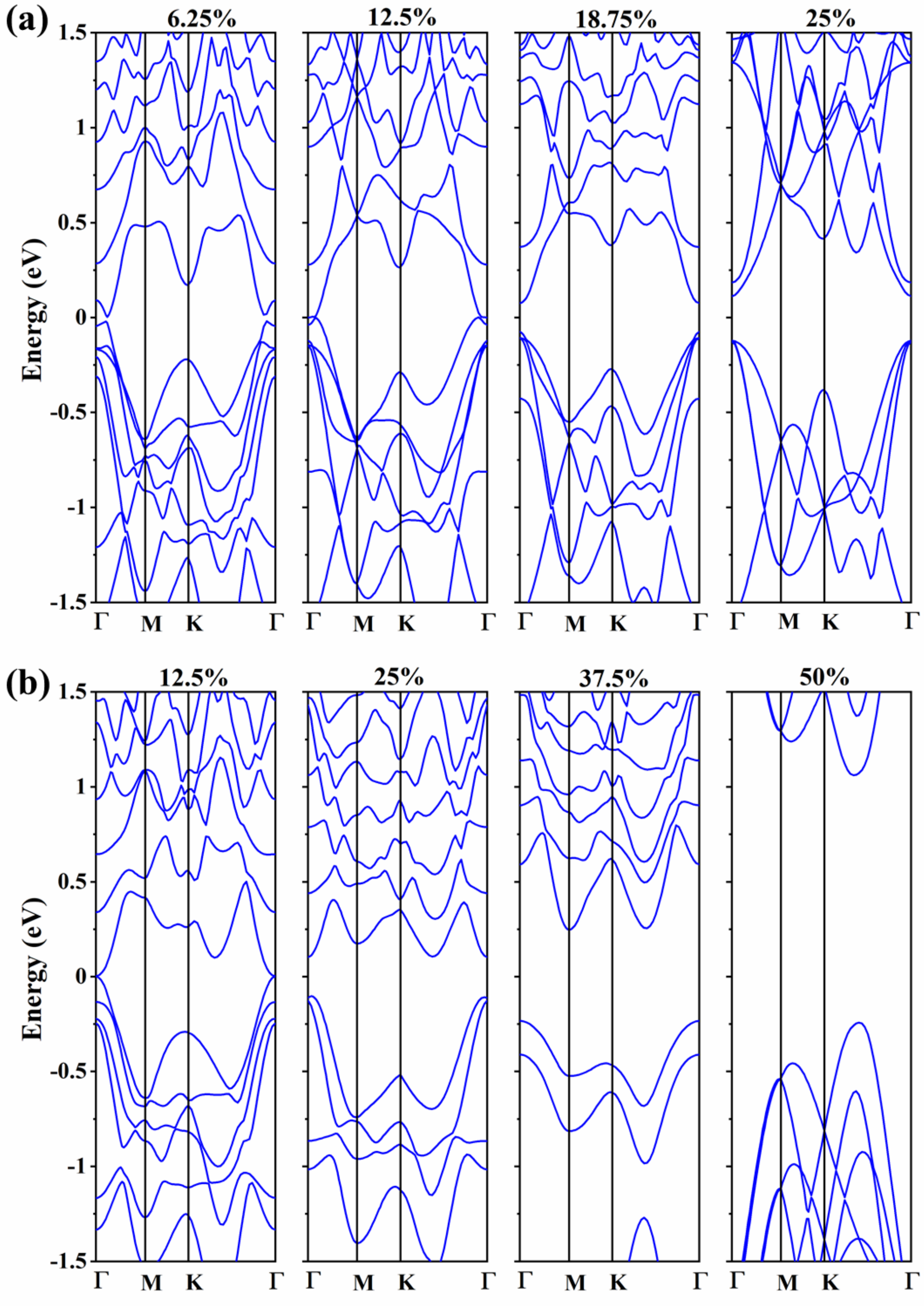

Figure S3 (a) Band structures of B doped bilayer C_3N with 1, 2, 3 and 4 B-atoms of case (i), respectively. (b) Band structures of B doped bilayer C_3N with 1, 2, 3 and 4 B-atoms of case (ii), respectively.

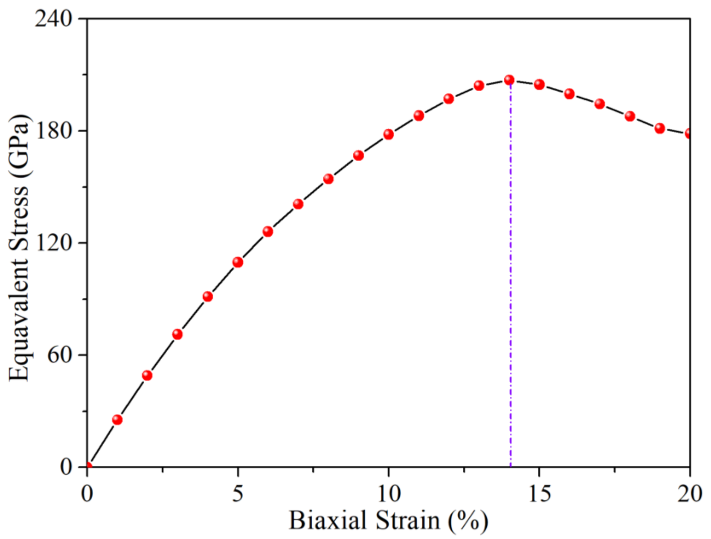

Figure S4 The strain-stress relation for monolayer C_3N. The strain is defined as $(a-a_0)/a_0$, where a_0 is the equilibrium lattice and a stands for strained one

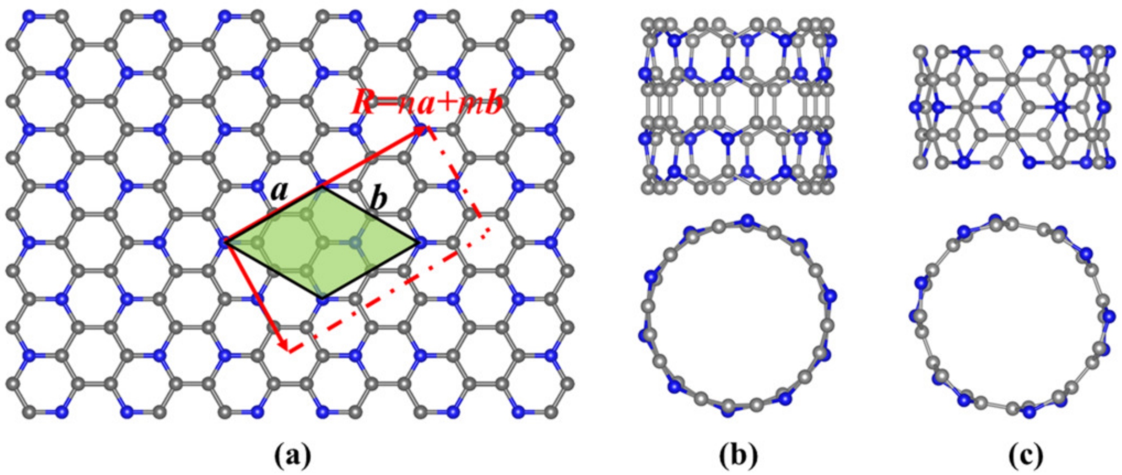

Figure S5 Schematic plots of C3N-nanotube which can be viewed as rolling up a C_3N sheet following the roll-up vector **R=na+mb**. Top and side view of (b) zigzag (5,0) and (c) armchair (3,3) tube.

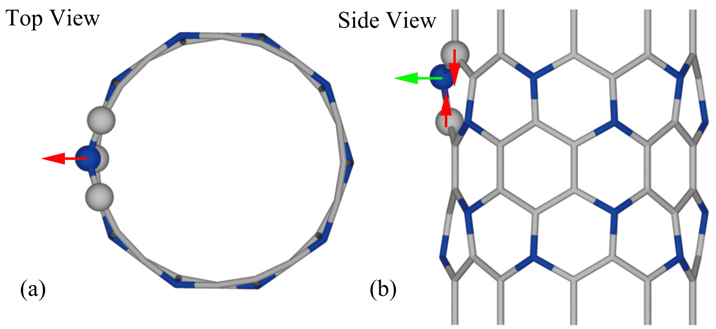

Figure S6 Top (a) and side (b) view of local structure of C_3N nanotube. The green arrow indicates the movement of N atom and red arrow presents the contraction of C-C distance.

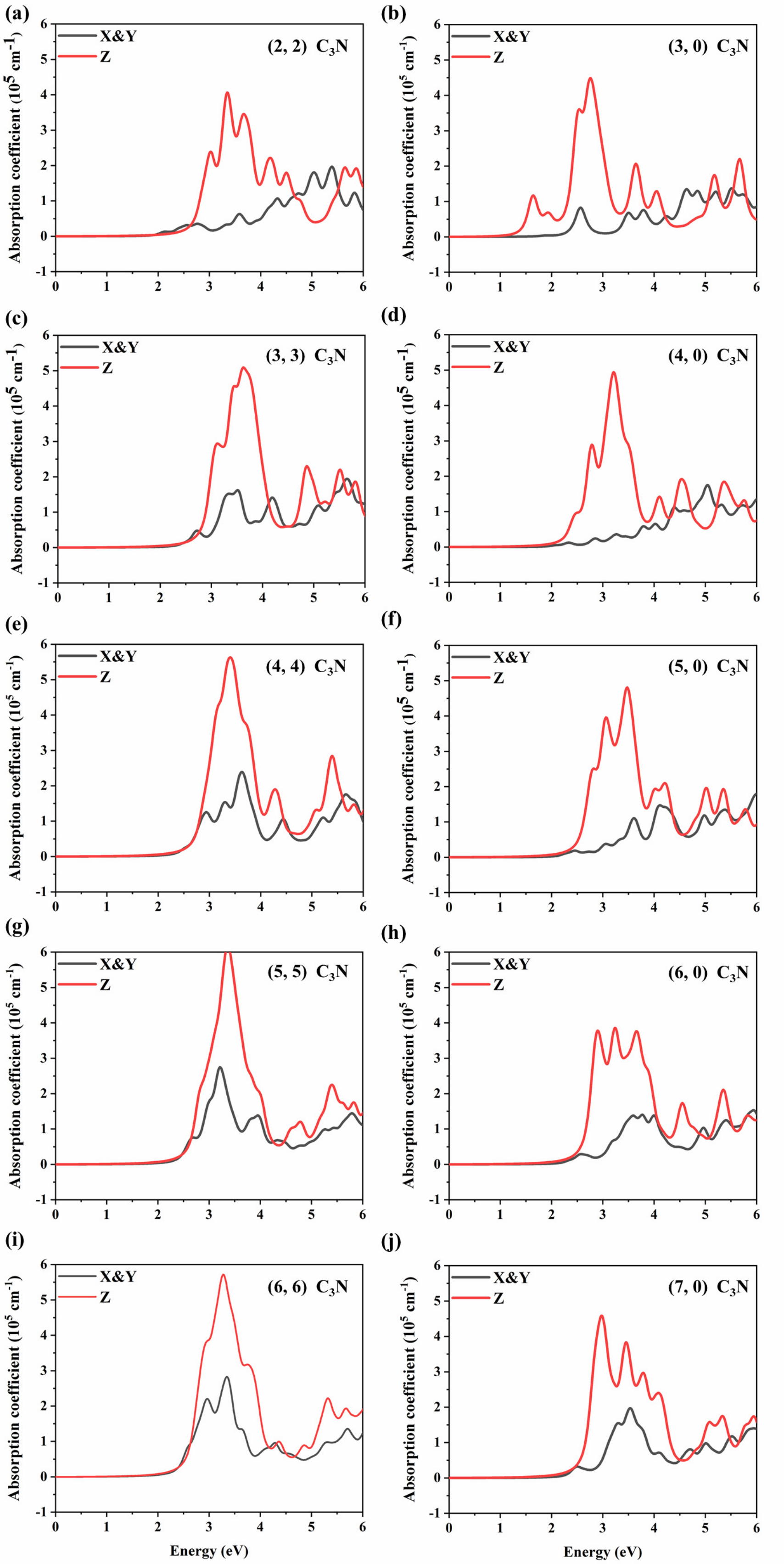

Figure S7 (a), (c), (e), (g)and (i) the optical absorption coefficients for armchair (2,2), (3,3), (4,4), (5,5) and (6,6) C_3N nanotube; (b), (d), (f), (h) and (j) the optical absorption cofficients for zigzag (3,0), (4,0), (5,0), (6,0) and (7,0) C_3N nanotube. The red line indicates the axial direction of the tube, and the black line indicates the radial direction of the tube.
